# Effect of thoracolumbar fascia injury on reported outcomes after percutaneous vertebroplasty

**DOI:** 10.3389/fsurg.2024.1379769

**Published:** 2024-05-16

**Authors:** Songbo Yang, Jie Tang, Zhaoyi Yang, Hongju Jin, Qinglei Wang, Huiming Wang

**Affiliations:** Department of Orthopedics, Beijing Geriatric Hospital, Beijing, China

**Keywords:** osteoporotic vertebral compression fracture, percutaneous vertebroplasty, thoracolumbar fascia injury, residual pain, patient-reported outcome measures

## Abstract

**Purpose:**

Thoracolumbar fascia injury is often associated with poor early pain relief after percutaneous vertebroplasty (PVP). This study will evaluate the effects of thoracolumbar fascia injury on early pain relief and time to get out of bed after PVP.

**Methods:**

A total of 132 patients treated with PVP for osteoporotic vertebral compression fractures (OVCF) were included and divided into injured group (52 cases) and non-injured group (80 cases) according to the existence of thoracolumbar fascia injury. Before surgery, 1 day, 3 days, 1 week, 1 month, and 3 months after surgery, and at the last follow-up, the primary patient-reported outcome measures (PROMs) were the visual analogue scale (VAS) of pain while rolling over and standing, and the secondary PROMs was the Oswestry disability index (ODI). Meanwhile, the achieved rate of minimal clinically important differences (MCID) and patient acceptable symptom states (PASS) of the above measures in both groups was evaluated at the last follow-up.

**Results:**

Except for the postoperative 3 months and the last follow-up, there were statistically significant differences in VAS-standing and ODI between the two groups at other time points after surgery (*P* < 0.05), and the non-injured group was significantly better than the injured group. At the last follow-up, there was no statistically significant difference in the MCID and PASS achievement rates of the above measures between the two groups (*P* > 0.05). In addition, the proportion of patients who got out of bed 1 and 3 days after surgery in the non-injury group was significantly higher than that in the injury group (*P* = 0.000 for both).

**Conclusion:**

Thoracolumbar fascia injury significantly affected early pain relief and extended time of getting out of bed after PVP. Attention should be paid to preoperative evaluation of thoracolumbar fascial injury in order to better predict the postoperative efficacy of PVP.

## Introduction

Osteoporotic vertebral compression fractures (OVCF) are the most common fragility fractures and can lead to severe back pain, limited mobility, kyphosis, sleep disturbances, reduced quality of life, and increased mortality (1). It is estimated that approximately 1.4 million patients are diagnosed with OVCF each year, which is the most common complication of osteoporosis (2). More recently, another epidemiological study found that the incidence of vertebral fractures among people aged 55–65 was 7.8 cases per 1,000 person-years. At age 75 or older, the incidence increases to 19.6 per 1,000 person-years in women and 5.2–9.3 per 1,000 person-years in men, respectively (3).

In clinical practice, most OVCF has resulted from minor injuries (e.g., fall to the floor, bending, stretching), or even occurs without any obvious trauma (e.g., cough and yawn). For elderly patients with osteoporosis, thoracic or lumbar magnetic resonance imaging (MRI) should be considered if they have significant low back pain (aggravated by rolling over and improved by bed rest), regardless of any significant history of trauma. At present, MRI is still the main method for diagnosing OVCF. In acute OVCF, vertebral bone marrow edema with low signal on T1WI, high signal on T2WI, and high signal on T2 weighted fat suppression image can be seen. Similar edema signals may exist in the thoracolumbar fascia and its dorsal subcutaneous tissue at the same time, suggesting that OVCF is complicated with thoracolumbar fascia injury (4). The incidence of thoracolumbar fascia injury in patients with acute OVCF has been reported to be 7.4%–45% (5–9). Thoracolumbar fascia for the protection and maintenance of spinal column. At the same time, it also participates in postural changes, load transfer and breathing movements (7, 10, 11). Histological studies have demonstrated the presence of nociceptive free nerve endings within the thoracolumbar fascia (11, 12). Experiments on human volunteers showed that noxious stimulation of the thoracolumbar fascia evokes pain. The human thoracolumbar fascia is more sensitive to chemical stimulations by hypertonic saline than the underlying erector spinae muscle and overlying subcutis (12). Furthermore, an inflammation or disorganization of the thoracolumbar fascia may contribute to chronic low back pain (12). Therefore, patients with OVCF thoracolumbar fascia injury have pain caused by thoracolumbar fascia injury in addition to the pain caused by vertebral fracture.

Patients with OVCF, whether with or without thoracolumbar fascia injury, should first choose conservative treatment, including bed rest, medication, exercise, and physical therapy, but often with limited effectiveness (6). If OVCF does not respond to conservative treatment, vertebral augmentation is an option (13). Percutaneous vertebroplasty (PVP) is now widely used and effective minimally invasive surgical procedure for OVCF. Bone cement is injected into the vertebra during the operation, and through the diffusion effect of the bone cement, it rims the trabecular bone of the vertebra to play an internal fixation role, thereby providing rapid pain relief and improving physiological function and quality of life. Compared with conservative treatment, PVP can significantly relieve pain and increase life expectancy in patients with OVCF (1, 8, 14, 15). Nevertheless, some patients still feel mild to moderate pain after PVP. In clinical exploration, we found that patients with thoracolumbar fascia injury in preoperative MRI examination often had poor pain relief in the early stage after PVP surgery, and the time of going to the ground was often late. It has also been reported in the literature that 4.6%–27.9% of the patients had some degree of residual pain after PVP (1, 8, 9, 13, 15–17). Yan et al. (7) found that there may be a certain relationship between preoperative thoracolumbar fascia injury and residual pain after PVP treatment for OVCF. Other studies have also found that OVCF combined with thoracolumbar fascia injury is one of the reasons for poor pain relief after vertebral augmentation (1, 8, 9, 13, 15–17). However, few studies have investigated the effect of preoperative thoracolumbar fascia injury on the time of getting out of bed after PVP. This study will retrospectively analyze prospective collected data to evaluate the effects of thoracolumbar fascia injury on early pain relief and time to get out of bed after PVP surgery, so as to better evaluate the prognosis before surgery and answer patients' queries.

## Materials and methods

### Patient selection

The medical records of patients with osteoporotic vertebral compression fractures who underwent percutaneous vertebroplasty in our hospital from March 2020 to March 2022 were retrospectively analyzed. This study was approved by the Ethics Review Committee of the Beijing Geriatric Hospital and followed procedures in accordance with the Declaration of Helsinki (TG2022-0041-09).

Inclusion criteria: (1) Single segment fresh thoracolumbar osteoporotic fracture (T6-L5), that is, the fractured vertebrae showed bone marrow edema signal on **MRI** T2 weighted fat suppression images; (2) L1–L4 bone mineral density *T* value of <−2.5 on dual-energy x-ray imaging; (3) Severe low back pain with a pain VAS score greater than 6 and no response to conservative treatment for at least a week; (4) No symptoms of nerve root and/or spinal cord compression; (5) Complete initial and follow-up data.

Exclusion criteria: (1) Fracture caused by high-energy trauma; (2) Pathological fractures, such as vertebral metastases or hemangiomas; (3) History of spinal surgery; (4) With Alzheimer's disease, Parkinson's disease, mental or psychological diseases; (5) Unable to get out of bed and move around before this fracture; (6) Incomplete case data; (7) Local infections or abnormal coagulation function.

**Figure 1:**
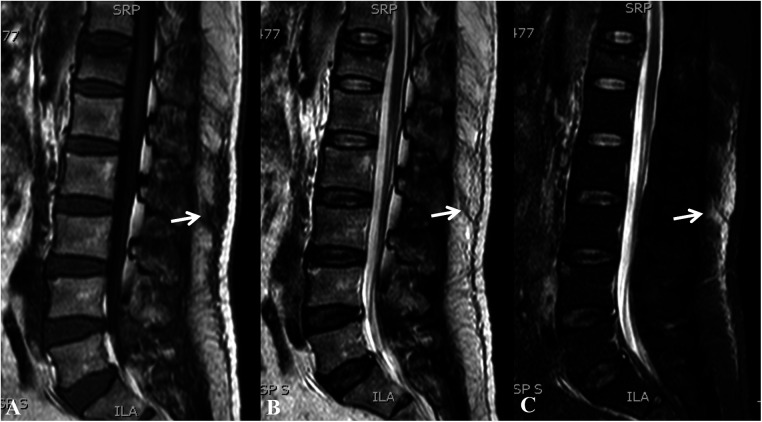
T12 vertebral compression fracture with thoracolumbar fascia injury (T12-S1) showed low signal intensity on T1 WI (**A**, arrow) and high signal intensity on T2 WI (**B**, arrow) and T2-STIR WI (**C**, arrow) on magnetic resonance imaging.

A total of 132 patients meeting the criteria were included and divided into injury group (52 cases) and non-injury group (80 cases) according to the presence of thoracolumbar fascia injury. Thoracolumbar fascia injury was defined based on MRI findings of fascia edema and focal obvious tenderness on physical examination in the corresponding level of fascia edema. In MRI examination, fascia edema showed high signal area in T2-weighted and T2-weighted fat suppression images and low signal area in T1-weighted images (Figure 1). In general, the thoracolumbar fascia injury is often in the shape of long strips or flakes on MRI, and involves multiple segments. It is easy to miss the diagnosis on T1 and T2-weighted images alone, but it is easy to find on T2 weighted fat suppression images.

Gender, age, body mass index (BMI), lumbar bone density *T* value, glucocorticoid usage, fracture age, fractured vertebra, type of injury and comorbidities were recorded before surgery. Among them, the fracture age refers to the time interval between fracture and PVP surgery. The vertebral fractures were recorded as thoracic, thoracolumbar, or lumbar fractures. The injury types were classified as obvious trauma, occult trauma or no obvious injury. Obvious trauma refers to the presence of a significant history of trauma, such as a flat fall or spinal impacted, while occult trauma refers to fractures caused by lifting heavy objects, lumbar sprains, and severe coughing. The types of comorbidities were recorded as the presence or absence of hypertension, diabetes, coronary heart disease, cerebral infarction, and chronic obstructive pulmonary disease. The comparison of the baseline characteristics of the two groups is shown in Table 1.

**Table 1:** Comparison of baseline characteristics between the two groups.

	Injured group (*n* = 52)	Non-injured group (*n* = 80)	*P* value
Female, *n* (%)	40 (76.9%)	65 (81.3%)	0.547*
Age (years)	72.31 ± 7.18	70.62 ± 6.57	0.166
BMD (T score)	−3.11 ± 0.47	−2.98 ± 0.51	0.142
BMI (kg/m^2^)	24.57 ± 2.93	25.02 ± 3.14	0.410
Glucocorticoid usage, *n* (%)	5 (9.6%)	9 (11.3%)	0.766*
Fracture age (day)	8.72 ± 3.37	9.38 ± 3.59	0.292
Fracture location, *n* (%)			0.863*
Thoracic	3 (5.8%)	5 (6.3%)	
Thoracolumbar	39 (75.0%)	56 (70.0%)	
Lower lumbar	10 (19.2%)	19 (23.8%)	
Types of spinal trauma, *n* (%)			0.335*
Obvious trauma	16 (30.8%)	17 (21.3%)	
Occult trauma	15 (28.8%)	21 (26.3%)	
No obvious trauma	21 (40.4%)	42 (52.5%)	
Concomitant disease, *n* (%)			0.628*
Hypertension	27 (45.8%)	36 (39.6%)	
Diabetes	9 (15.3%)	19 (20.9%)	
CHD	8 (13.6%)	17 (18.7%)	
Cerebral infarction	13 (22.0%)	14 (15.4%)	
COPD	2 (3.4%)	5 (4.7%)	

*P*-values from Student's *t*-test.

BMD, bone mineral density; BMI, body mass index; CHD, coronary heart disease; COPD, chronic obstructive pulmonary disease.

^*^ *P*-values from *χ*^2^test.

### Surgical procedure

All patients underwent local infiltration anesthesia and unilateral pedicle PVP surgery. The patient is placed in a prone position and put on pillows in front of their shoulders, chest, and iliac spine, straightening the thoracolumbar spine and suspending the abdomen. Based on physical examination and imaging data, the injured vertebrae were identified and C-arm fluoroscopy was used to locate the vertebra during the operation. After routine disinfection, 1% lidocaine was used for layer-by-layer anesthesia. Under C-arm fluoroscopy, the tip of the puncture needle was placed on the outer upper edge of the pedicle projection and hammered into the needle. Then, the needle with the core is drilled into the front third of the pedicle, ensuring that it does not enter the spinal canal. At this time, pull out the inner core of the puncture needle, insert the guide needle and pull out the puncture needle sleeve. After the guide needle is drilled into the vertebral body, it is placed into the working channel along the guide needle. At this time, under the fluoroscopy of the C-arm machine, ensure that the tip of the needle reaches the shadow of the spinous process when in the anterioposterior position and the anterior part of the vertebral body when in the lateral position. At the same time, the guide needle was rotated and removed, and the polymethylmethacrylate (PMMA) bone cement, which had been adjusted into “toothpaste shape” and was in the late stage of threading, was injected along the working channel under continuous fluoroscopy. After satisfactory injection of PMMA bone cement, the working cannula was pulled out and the surgical incision was sutured. After observation of complete hardening of PMMA bone cement, the patient was sent back to the ward in supine position. The time of operation, the amount of bone cement and leakage were recorded during the operation, and systematic anti-osteoporosis treatment was performed after the operation.

### Patient-reported outcome measures (PROMs)

Present study used PROMs to assess the benefits of treatment. Patient-reported outcome measure is any report about a patient's health that comes directly from the patient and does not require an evaluation by a clinician or anyone else. At preoperative, postoperative 1 day, 3 days, 1 week, 1 month, 3 months, and the last follow-up, the visual analogue scale (VAS) was used to assess the degree of thoracolumbar pain rolling over and standing, and the Oswestry disability index (ODI) was used to assess the patient's functional impairment. The VAS score consists of a 100 mm line with one end of the line representing “completely painless” (0 points) and the other end representing “extreme pain” (10 points). The patient is asked to mark the appropriate place on the line to indicate the level of pain. The Oswestry disability index is composed of 10 items that reflect the patient's lower back and leg pain, sleep, sexual activity, etc. Each item has a minimum score of 0 and a maximum score of 5. The higher the score, the more severe the dysfunction. Ask patients to rate each of these 10 items separately, and then accumulate them to obtain the final score of the patient. The percentage of the highest total score (50 points) is the Oswestry disability index. The difference score (*Δ*) was calculated by subtracting the preoperative score from the score at the last follow-up. Patients with minimal clinically important differences (MCID) at the last follow-up were defined according to the criteria defined in previous studies: ΔODI was −9.5 (18) and ΔVAS was −2.2 for pain when rolling over and standing (19). The thresholds for defining patient acceptable symptom states (PASS) were 3 points (VAS score) (20) and 22 points (ODI index) (21).

In addition, referring to the methods proposed by Si et al. (22), the criteria for determining whether patients can get out of bed and move are as follows: if the standing VAS score is less than 5, patients should get out of bed; If the VAS score of standing position is greater than or equal to 5 points, the patient should be told to continue in bed and undergo physiotherapy, and the low back muscle function exercise in bed state is encouraged. When the pain VAS score is less than 5 in the standing position, then get out of bed and walk. The VAS scores at the time of turning over and standing and the number of patients allowed to get out of bed on the day 1, 3 and 7 after surgery were compared between the two groups to analyze the influence of thoracolumbar fascia injury on the early postoperative treatment effect. Non-steroidal anti-inflammatory drugs (NSAIDs) were not routinely administered after surgery, but only when the patient had obvious pain (VAS score greater than or equal to 4 points), NSAIDs were temporarily administered, and the VAS score before administration was recorded.

### Statistical analysis

Statistical analyses were carried out using SPSS 23.0 (SPSS Inc., Chicago, IL, USA). When comparing the baseline data and the MCID and PASS attainment rates between the two groups, Chi-square test was applied to the classified data, which was expressed as count (percentage). Independent sample t test was used for continuous data with normal distribution, expressed as mean ± standard deviation (SD). VAS-turning over, VAS-standing and ODI was compared between groups at different time points of follow-up by two-way repeated measure ANOVA. Statistical significance for analyses in this study was set at a *P*-value < 0.05.

## Results

### General characteristics

All patients successfully completed the operation without serious complications. There was no significant difference in operation time between injured group (31.49 ± 5.27 min) and non-injured group (30.12 ± 4.85 min) (*P* = 0.128). The dosage of bone cement was 3.56 ± 0.42 ml in the injured group and 3.67 ± 0.53 ml in the non-injured group, and there was no statistical difference between the two groups (*P* = 0.163). A total of 14 cases (10.6%) of bone cement leakage occurred in the two groups, 5 cases (9.6%) in the injured group and 9 cases (11.3%) in the non-injured group, with no statistically significant difference (*P* = 0.766). The follow-up time of the injured group was 17.62 ± 5.54 months, and that of the non-injured group was 19.01 ± 5.72 months, which showed no significant difference between the two groups (*P* = 0.170). During the follow-up period, a total of 13 cases (9.8%) suffered secondary adjacent vertebral fractures, including 8 cases (15.4%) in the injured group and 5 cases (6.3%) in the non-injured group. There was no significant difference in the incidence of secondary adjacent vertebral fractures between the two groups (*P* = 0.085), and symptoms improved after PKP again.

### Therapeutic effect evaluation

There were no statistically significant differences in baseline PROMs between the two groups (Table 2). The VAS-rolling score, VAS-standing score and ODI of the two groups at each time point after surgery were significantly higher than those before surgery, with statistical significance (*P* < 0.05, Table 2). There was no statistical significance in VAS-rolling score between the two groups at different time points after surgery (*P* > 0.05), as shown in Table 2. However, except for the postoperative 3 months and the last follow-up, there were statistically significant differences in VAS-standing and ODI between the two groups at other time points after surgery (*P* < 0.05, Table 2), and the non-injured group was significantly better than the injured group. At the last follow-up, there was no statistically significant difference in the MCID and PASS achievement rates of VAS-rolling score, VAS-standing score, and ODI between the two groups (*P* > 0.05), as shown in Table 3.

**Table 2:** Comparison of VAS score when turning over and standing up before and after treatment and ODI between the two groups.

	Injured group (*n* = 52)	Non-injured group (*n* = 80)	*P* value
VAS score when turning over			
Preoperative	7.52 ± 0.53	7.41 ± 0.44	0.198
1 day after surgery	4.19 ± 0.72*	3.96 ± 0.69*	0.068
3 days after surgery	3.21 ± 0.57*	3.05 ± 0.61*	0.133
1 week after surgery	2.32 ± 0.49*	2.19 ± 0.51*	0.149
1 month after surgery	2.03 ± 0.31*	1.94 ± 0.36*	0.141
3 months after surgery	1.76 ± 0.41*	1.68 ± 0.34*	0.226
Last follow-up	1.69 ± 0.28*	1.65 ± 0.32*	0.463
VAS score while standing up			
Preoperative	7.85 ± 0.61	7.69 ± 0.57	0.128
1 day after surgery	4.33 ± 0.76*	3.97 ± 0.70*	**0** **.** **006**
3 days after surgery	3.72 ± 0.66*	3.08 ± 0.68*	**0** **.** **000**
1 week after surgery	3.34 ± 0.58*	2.61 ± 0.47*	**0** **.** **000**
1 month after surgery	3.88 ± 0.49*	2.43 ± 0.32*	**0** **.** **000**
3 months after surgery	2.21 ± 0.34*	2.14 ± 0.29*	0.208
Last follow-up	2.05 ± 0.32*	1.97 ± 0.25*	0.111
ODI, %			
Preoperative	71.39 ± 7.58	69.86 ± 7.71	0.264
1 day after surgery	39.25 ± 6.24*	28.77 ± 7.47*	**0** **.** **000**
3 days after surgery	36.51 ± 6.38*	26.19 ± 6.25*	**0** **.** **000**
1 week after surgery	34.62 ± 6.11*	25.08 ± 5.82*	**0** **.** **000**
1 month after surgery	30.29 ± 5.77*	24.27 ± 5.61*	**0** **.** **000**
3 months after surgery	25.38 ± 4.41*	24.17 ± 4.82*	0.148
Last follow-up	21.42 ± 2.83*	21.06 ± 2.65*	0.459

Data are presented as mean ± SD.

VAS, visual analogue scale; ODI, Oswestry disability index.

Bold values means the statistically significant values.

* Compared to preoperative, *P* < 0.05.

**Table 3:** The proportion of VAS score when turning over and standing up and ODI reaching MCID and PASS.

	Injured group (*n* = 52)	Non-injured group (*n* = 80)	*P* value
MCID VAS-turning over			0.704
No	7 (13.5%)	9 (11.3%)	
Yes	45 (86.5%)	71 (88.8%)	
MCID VAS-standing up			0.834
No	9 (17.3%)	15 (18.8%)	
Yes	43 (82.7%)	65 (81.3%)	
MCID ODI			0.681
No	14 (26.9%)	19 (23.8%)	
Yes	38 (73.1%)	61 (76.3%)	
PASS VAS-turning over			0.723
No	9 (17.3%)	13 (15.0%)	
Yes	43 (82.7%)	67 (85.0%)	
PASS VAS-standing up			0.855
No	11 (21.2%)	18 (22.5%)	
Yes	41 (78.8%)	62 (77.5%)	
PASS ODI			0.751
No	13 (25.0%)	22 (27.5%)	
Yes	39 (69.4%)	58 (72.5%)	

Data are expressed as a count (%). *P*-values from *χ*^2^test.

VAS, visual analog scale; ODI, Oswestry disability index; MCID, minimal clinically important differences; PASS, patient acceptable symptom states.

The number of patients in the injured group who could get out of bed and walk on 1, 3 and 7 days after surgery was 15, 33 and 49, respectively, while the number in the non-injured group was 59, 77 and 80, respectively. The proportion of patients who got out of bed 1 and 3 days after surgery in the non-injury group was higher than that in the injury group, and the difference was statistically significant (*P* = 0.000 for both). However, after clinically relevant treatment, all patients could get out of bed and move on the 7th day after surgery, as shown in Table 4.

**Table 4:** Comparison of the number of patients who met the standard of getting out of bed at 1, 3 days and 1 week after surgery between the two groups.

	Injured group (*n* = 52)	Non-injured group (*n* = 80)	*P* value
1 day after surgery, *n* (%)	15 (28.8%)	59 (73.8%)	**0** **.** **000**
3 days after surgery, *n* (%)	33 (63.5%)	77 (96.3%)	**0** **.** **000**
1 week after surgery, *n* (%)	49 (94.2%)	80 (98.8%)	0.300

Data are expressed as a count (%). *P*-values from *χ*^2^test.

Bold values means the statistically significant values.

## Discussion

Osteoporotic vertebral compression fractures are a common cause of low back pain in the elderly population, with approximately 1.4 million new cases worldwide each year (2). Conservative treatment should be the first choice for OVCF. If conservative treatment does not respond, PVP is an effective minimally invasive treatment for rapid pain relief and early mobilization (1, 8, 13–15). Evidence-based guidelines (23) and systematic reviews (24) conclude that there is moderate evidence that PVP can be used to treat patients with symptomatic OVCF refractory to conservative treatment. Despite this, some patients still experience mild to moderate residual pain after PVP.

Due to different inclusion criteria and evaluation time points, 4.6%–27.9% of OVCF patients reported early residual pain after PVP (1, 8, 9, 13, 15–17). Among them, the study carried out by Yang et al. was the largest sample size included in the literature and retrospectively analyzed 1,316 patients with OVCF treated with PVP. They defined residual pain after PVP as a VAS score greater than 4 points immediately after surgery and 1 month after surgery. Residual pain was found in 60 cases (4.6%). Meanwhile, univariate and multivariate analysis showed thoracolumbar fascia injury is a strong risk factor for residual pain after PVP, with an OR of 3.805 (*P* = 0.002) (8). Other studies have also found that thoracolumbar fascia injury is a risk factor for residual low back pain after vertebral augmentation (including PVP and percutaneous kyphoplasty), with OR values of 4.083 (*P* = 0.032) (15), 4.11 (*P* = 0.014) (9), and 11.377 (*P* < 0.001) (13), respectively. However, Yu et al.'s study found that thoracolumbar fascia injury was not associated with postoperative residual pain (*P* = 0.31). (17) Our retrospective cohort study found that VAS-standing scores and ODI scores in the thoracolumbar fascia injury group were worse than those in the non-injury group at all time points within 1 month after surgery (*P* < 0.05). However, there was no significant difference between the two groups at 3 months and the last follow-up (*P* > 0.05). For OVCF patients with thoracolumbar fascia injury, lumbar muscle exercise and physiotherapy can be performed. The patient can be informed that with the repair of the thoracolumbar fascia injury and the regression of edema, the residual low back pain in the early postoperative period will also be relieved. This also explains why VAS-standing score and ODI were significantly different between the two groups only in the early postoperative period (1 day, 3 days, 1 week, and 1 month after surgery). In addition, Ourt study also found that there were no significant differences in VAS-turning scores between the two groups at each time point (*P* > 0.05). These results indicated that the injury of thoracolumbar fascia affected the early pain and functional improvement after PVP. Therefore, thoracolumbar fascia injury could be an alternative overlooked cause of residual back pain after PVP (7, 8).

In the previous prospective study, Yan et al. included 133 patients with OVCF and used VAS score and Chinese modified Oswestry Disability Index to evaluate the impact of thoracolumbar fascia injury on PVP treatment of OVCF. The results showed that postoperative VAS and ODI scores in the injured group were worse than those in the non-injured group (7). Our results are similar to those of Yan et al. But in our study, we evaluated VAS scores for standing up vs. rolling over, as well as specific time to get out of bed. At the last follow-up, MCID and PASS were used for the first time to evaluate whether the changes in VAS and ODI scores before and after surgery had clinical significance. Our study found that at the last follow-up, there was no significant difference in the MCID and PASS achievement rates of VAS-rolling score, VAS-standing score, and ODI between the two groups (*P* > 0.05). These results indicated that the injury of thoracolumbar fascia did not affect the medium and long-term effects of PVP. In addition, our research also found the proportion of patients who got out of bed 1 and 3 days after surgery in the non-injury group was higher than that in the injury group (*P* = 0.000 for both). However, after clinically relevant treatment, all patients could get out of bed and move on the 7th day after surgery. These results suggest that thoracolumbar fascia injury affects the early time of getting out of bed after PVP. In other words, patients with thoracolumbar fascia injury should be advised that they may need to delay getting out of bed after PVP.

There are also various forms of nomenclature for thoracolumbar fascial injuries in the literature, such as posterior fascia oedema (9) and lumbodorsal fascia contusion (8). Thoracolumbar fascia injury is common among patients with OVCF. In the prospective cohort study reported by Yan et al, the prevalence of thoracolumbar fascial injury was as high as 42.1% (7). However, another retrospective study found that the incidence of thoracolumbar fascia injury was 28.3% in the satisfied group and as high as 43% in the dissatisfied group (8). Although most of the OVCFs resulted from low energy impacts or were even caused by occult trauma, the prevalence of thoracolumbar fascia injury was 7.4%–45% (5–9).

The thoracolumbar fascia is a girdling structure comprising a complex arrangement of several aponeurotic and multiple fascial layers that separate the paraspinal muscles from the muscles of the posterior abdominal wall. In the lumbar region, the thoracolumbar fascia is composed of three distinct layers: posterior, middle, and anterior layers. The posterior layer originates medially from the tip of the spinous processes of the lumbar vertebrae and the supraspinous ligament; a superficial lamina is the aponeurosis of the latissimus dorsi, and a deep lamina covers the posterior surface of the paraspinal muscles. The middle layer is attached to the tips of the transverse processes of the lumbar vertebrae and extends laterally behind the quadratus lumborum. The anterior layer covers the anterior surface of the quadratus lumborum and is attached medially to the transverse processes of the lumbar vertebrae behind psoas major (25, 26). The “hydraulic amplification” effect produced by thoracolumbar fascia is the key mechanism of the formation of paravertebral muscle strength. With the continuation of latissimus dorsi and transversalis abdominis, it can integrate the mechanical action of the limbs, abdomen and spine. This is an important part of the paravertebral stability structure of the spine (25). The thoracolumbar fascia plays a key role in general locomotion of the body meaning that it facilitates stabilizing the body, generating and releasing the forces required by the body to move effectively such that an individual is able to ambulate normally without falling. Since thoracolumbar fascia is used frequently during an entire day hence it is prone to overuse and hence various injuries to it resulting in thoracolumbar fascia pain (10, 25).

Clinically, OVCF patients are often accompanied by thoracolumbar fascia injury, and the two often co-occur. In our retrospective study, 39.4% of OVCF patients were accompanied by thoracolumbar fascia injury, which may be related to the type of spinal injury. It is necessary to further expand the sample size to explore the specific correlation. The injury of thoracolumbar fascia in OVCF patients is mainly in the posterior layer, and the injury of fascial complex during torsion resistance and load transfer can cause pain. Pain caused by thoracolumbar fascia injury is usually masked by acute and severe pain caused by OVCF, which also explains why there was no significant difference in VAS before surgery between the two groups in this study, but there was a significant difference in the early postoperative period. Although PVP can restore vertebral stiffness and effectively improve the severe pain caused by vertebral fractures, it cannot decompress the thoracolumbar fascia or improve the severe inflammatory response of the thoracolumbar fascia. At the same time, it could not stabilize the thoracolumbar fascia and restore the function of the thoracolumbar fascia conducting load. Therefore, PVP cannot fundamentally relieve the pain caused by thoracolumbar fascia injury, resulting in residual pain. As fascia-like ligaments-has a much less abundant blood supply than muscles, it can be expected to heal as slowly as ligaments, and may therefore be a more likely facilitator of chronic back pain than injured muscles (27). Some studies have found that it may take more than 1 month to completely reverse the thoracolumbar fascia abnormality (28). In addition, during follow-up, we found that residual pain persisted in some patients with thoracolumbar fascia injury, often lasting more than 3 months. MRI examination showed that the injury of thoracolumbar fascia was significantly worse than that before surgery. It can be seen that some patients with thoracolumbar fascia injury cannot heal itself, and even aggravate, which may be an important cause of sustained residual pain after surgery.

The thoracolumbar fascia is rich in nerve endings (11, 25, 29). A study investigating the distribution and density of CGRP-positive fibers in different tissues reported a three times higher density in the thoracolumbar fascia than in the spinal muscles (30). Most CGRP- and SP-ir (sensory) fibers are located in the outer layer of the fascia and the subcutaneous tissue (31). Therefore, the thoracolumbar fascia may well be an important source for low back pain. A comparison between an inflamed and intact thoracolumbar fascia showed an increase of the CGRP- and SP-positive fibre in the inflamed thoracolumbar fascia, which may explain the pain from a pathologically altered fascia (11). In addition, the human thoracolumbar fascia is more sensitive to chemical stimulations by hypertonic saline than the underlying erector spinae muscle and overlying subcutis, according to various pain measures (peak pain, pain duration, pain distribution, and affective pain descriptors). Furthermore, an inflammation or disorganization of the thoracolumbar fascia may contribute to chronic low back pain (12). The thoracolumbar fascia may generate back pain in three possible ways (32). First, if you sustain micro-injuries and/or inflammation-often the two are related-these may stimulate changes in the free nerve endings that live in the thoracolumbar fascia. Second, after an injury, it's common for tissues to become stiff. It is unclear if this change is the cause or the result of having back pain, but alterations of the quality of thoracolumbar fascia have been noted in some studies of patients with back pain. Finally, as we saw above, injury can irritate nerves, which can lead to increased sensitivity to pain.

Patients with osteoporotic vertebral compression fracture should not only pay attention to the treatment of the fractured vertebral body, but also pay attention to the role of the thoracolumbar fascia of the back. Good function of thoracolumbar fascia can reduce the bed time after PVP and has important significance in improving the early curative effect of surgery. Si et al. (22) believed that if there were high T1 signal and low T2 signal in the thoracolumbar fascia on preoperative MRI, accompanied by high FSE signal, it could be diagnosed as thoracolumbar fascia injury and it was necessary to delay getting out of bed. At the same time, physiotherapy, function exercise, drug analgesia and other treatments should be carried out for thoracolumbar fascia injury during perioperative period to promote the recovery of thoracolumbar fascia function, shorten the bed time and get out of bed as soon as possible, so as to reduce the risk of various complications.

There are some potential limitations to our study that need to be mentioned. First, the current study did not evaluate the specific injury type and severity of thoracolumbar fascia, and there are many risk factors for residual pain after PVP [(8, 15, 17)], and preoperative thoracolumbar fascia injury is only one of them. Further studies are needed to explore the effect of these factors on postoperative PVP. Second, although we believe that the differences in VAS-standing score and ODI between the two groups can be attributed to the presence of thoracolumbar fascia injury, there are many variables that may confound the results, and further multivariate analysis of a large sample is required to eliminate confounding variables to validate our findings. Finally, this study is a retrospective review of prospective data collection, and selection bias may be present. Further prospective studies may more accurately reflect the effect of thoracolumbar fascia injury on PVP treatment.

## Conclusions

This study indicates that thoracolumbar fascia injury significantly affected early pain relief and extended time of getting out of bed after PVP. In OVCF patients, attention should be paid to evaluating whether there is damage in the thoracolumbar fascia before surgery, so as to better predict the postoperative efficacy of PVP and answer the prognosis inquiry of patients.

## Data Availability

The original contributions presented in the study are included in the article/Supplementary Material, further inquiries can be directed to the corresponding author.
